# Supplementary material for: Global declines in net primary production in the ocean color era
Source: Nat Commun. 2025 Jul 1;16:5821. doi: 10.1038/s41467-025-60906-y (PMC12219224; doi:10.1038/s41467-025-60906-y)
Supplement: Supplementary file 1 — Supplementary Information [file 41467_2025_60906_MOESM1_ESM.pdf]

Supplementary Material for:

'Global declines in net primary production in the ocean color era'

Greg M. Silsbe[1]*, James Fox[2]*, Toby K. Westberry[2] and Kimberly H. Halsey[2]

[1]Horn Point Laboratory, University of Maryland Center for Environmental Science. Cambridge MD, United States.

[2]Oregon State University. Corvallis OR, United States.

*These authors contributed equally to this work.

Corresponding authors: Greg Silsbe (gsilsbe@umces.edu), James Fox (james.fox@osucascades.edu)

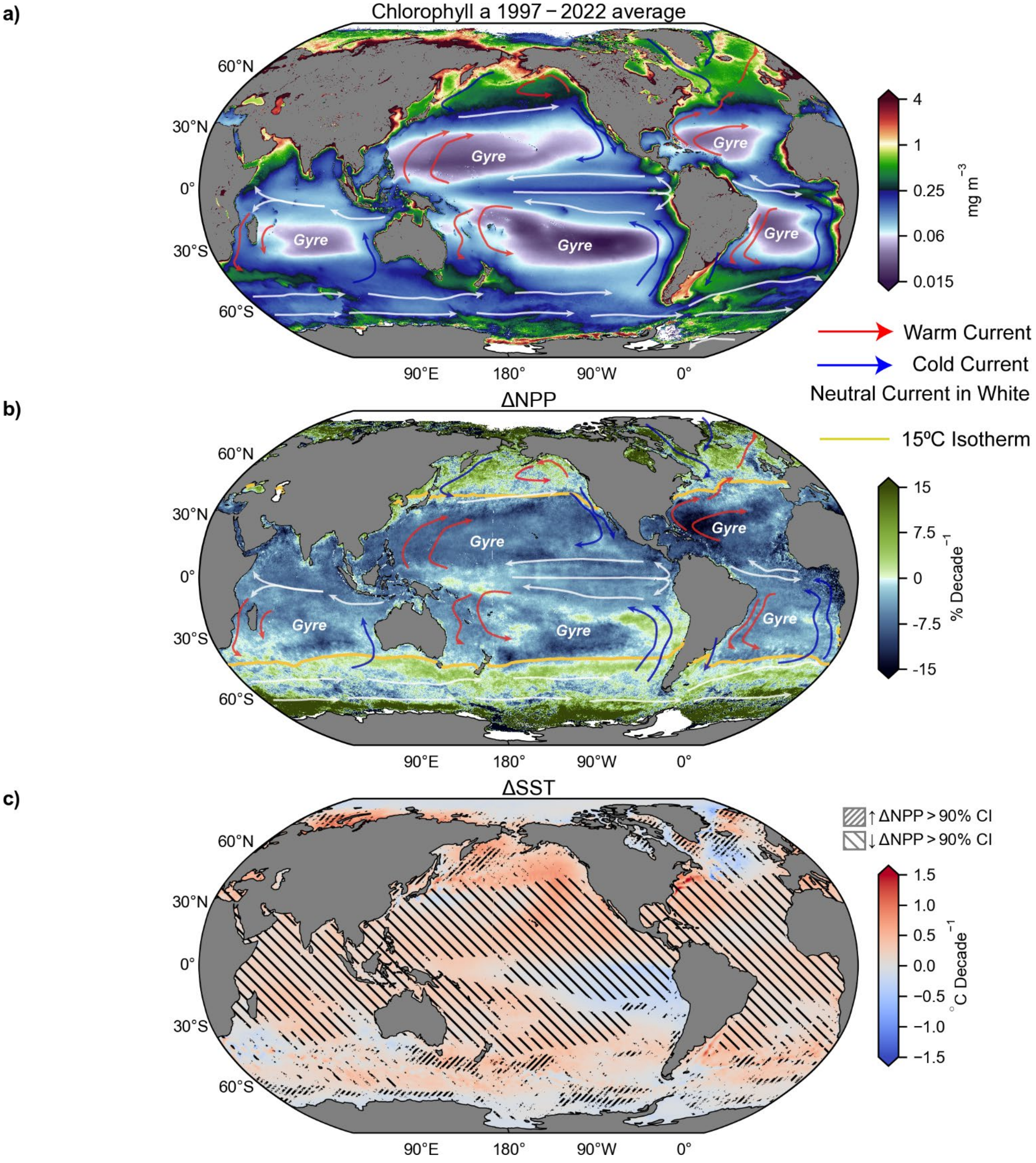

Supplementary Figure 1: **Spatial patterns of chlorophyll and net primary production (NPP) and sea surface temperature (SST) trends alongside major ocean currents. a**. Average monthly chlorophyll (Sep-1997 to Dec-2022). **b.** Normalized decadal NPP trends. Panels a and b highlights major ocean gyres, warm (red), neutral (white), and cool (blue) surface currents, the average 15°C isotherm (yellow) across the same period is shown in Panel B. **c.** SST trends (Sep-1997 to Dec-2022) where vertical hatching denotes regions of statistically increasing (forward) and decreasing (backward) NPP trends.

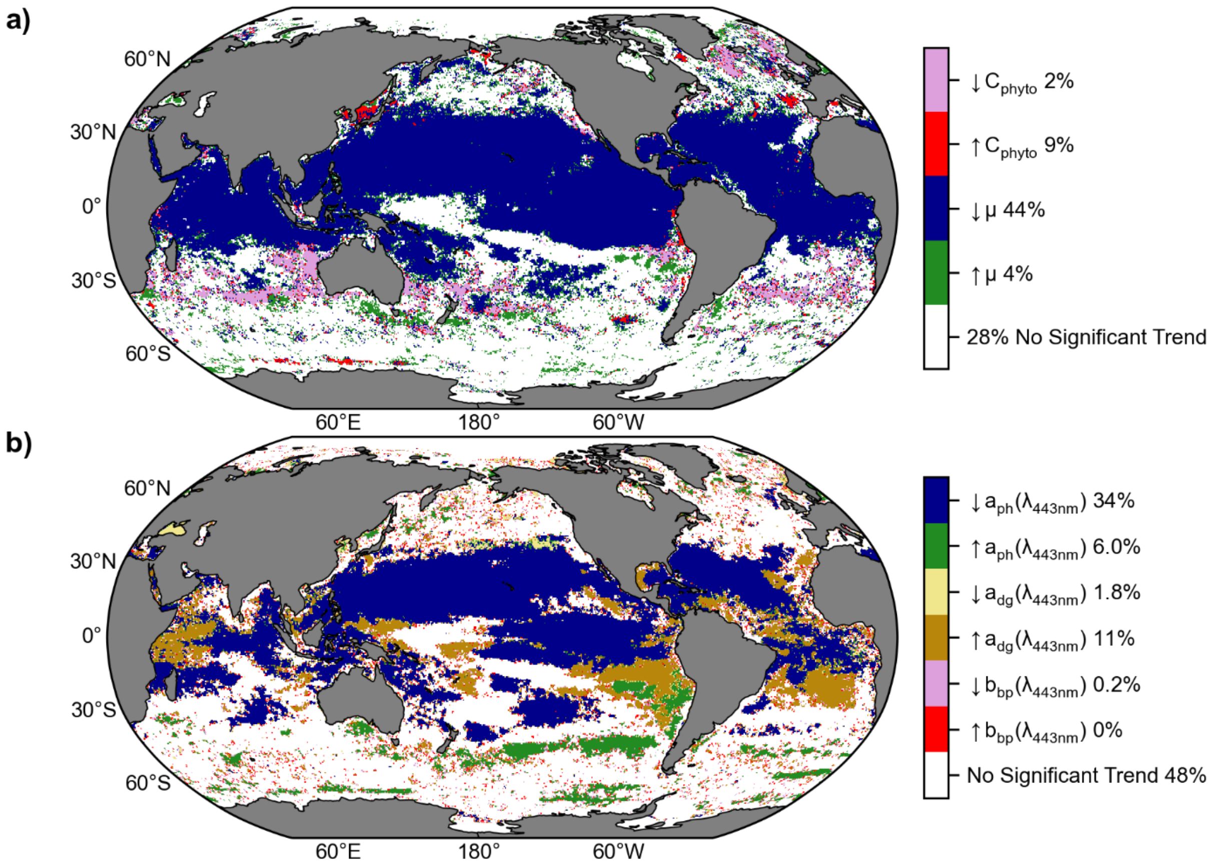

Supplementary Figure 2: **Decadal trends in phytoplankton growth rates (μ) and carbon biomass (C_{phyto})**. **a.** Frequency map showing the largest statistically significant normalized trend (increasing or decreasing) for euphotic zone integrated C_{phyto} and μ. **b.** Frequency map showing the largest statistically significant normalized trend (increasing or decreasing) for phytoplantkon absorption coefficient (a_{ph}), the absorption coefficient of detrital and dissolved organic matter (a_{dg}), and particulate backscattering (b_{bp}), all inherent optical properties are evaluated at 443 nm.

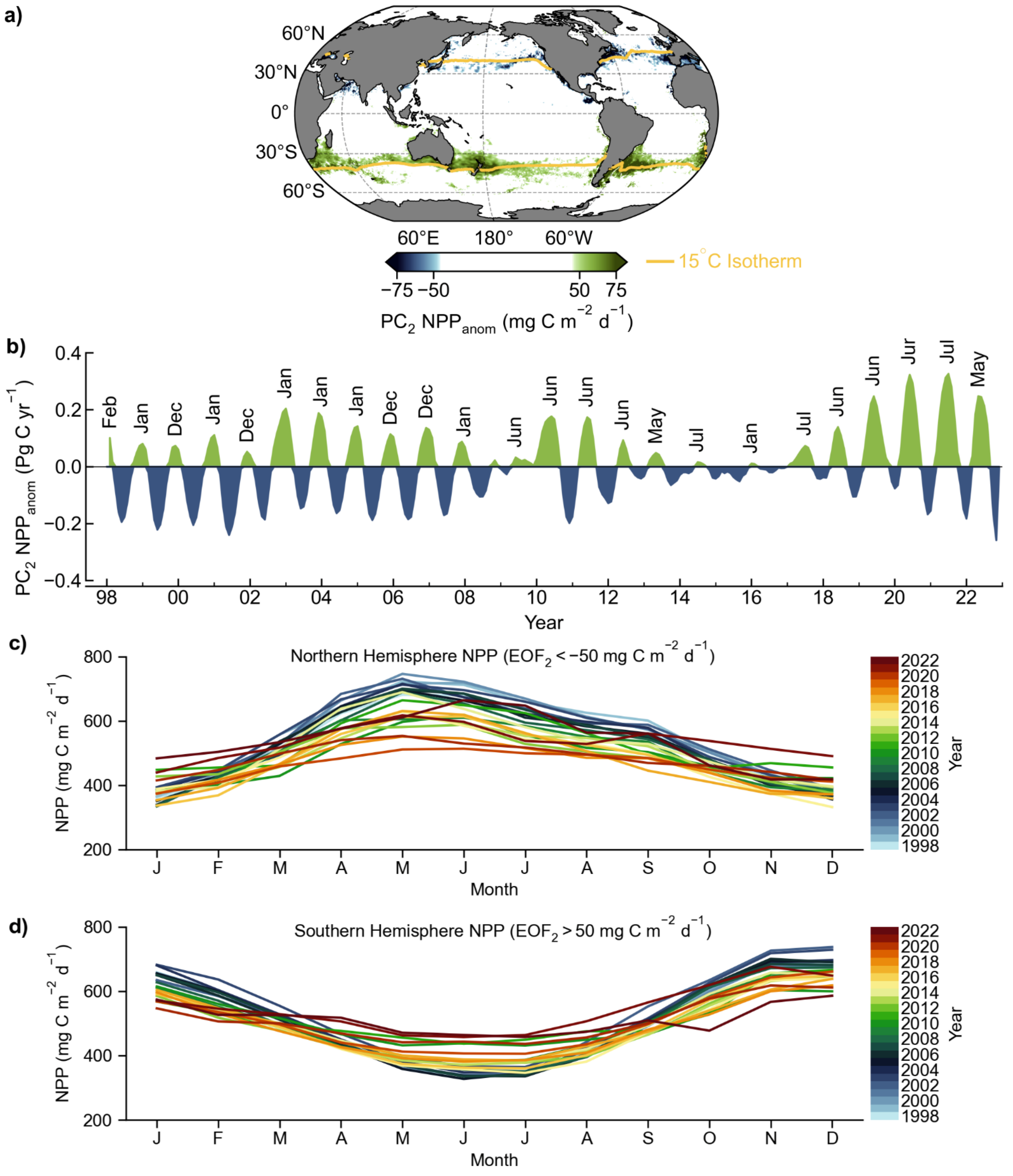

Supplementary Figure 3: Shifting seasonality in net primary production (NPP). **a.** The spatial component of the second principal component of NPP anomalies highlighting regions of strong variance alongside the mean 15°C sea surface temperature (SST) isotherm. **b.** The corresponding time component with peak months labeled. **c.** and **d.** Spatially averaged NPP in the northern and southern hemispheres respectively for regions where $|PC_2| > 50$ mg C m^{-2} day^{-1}.

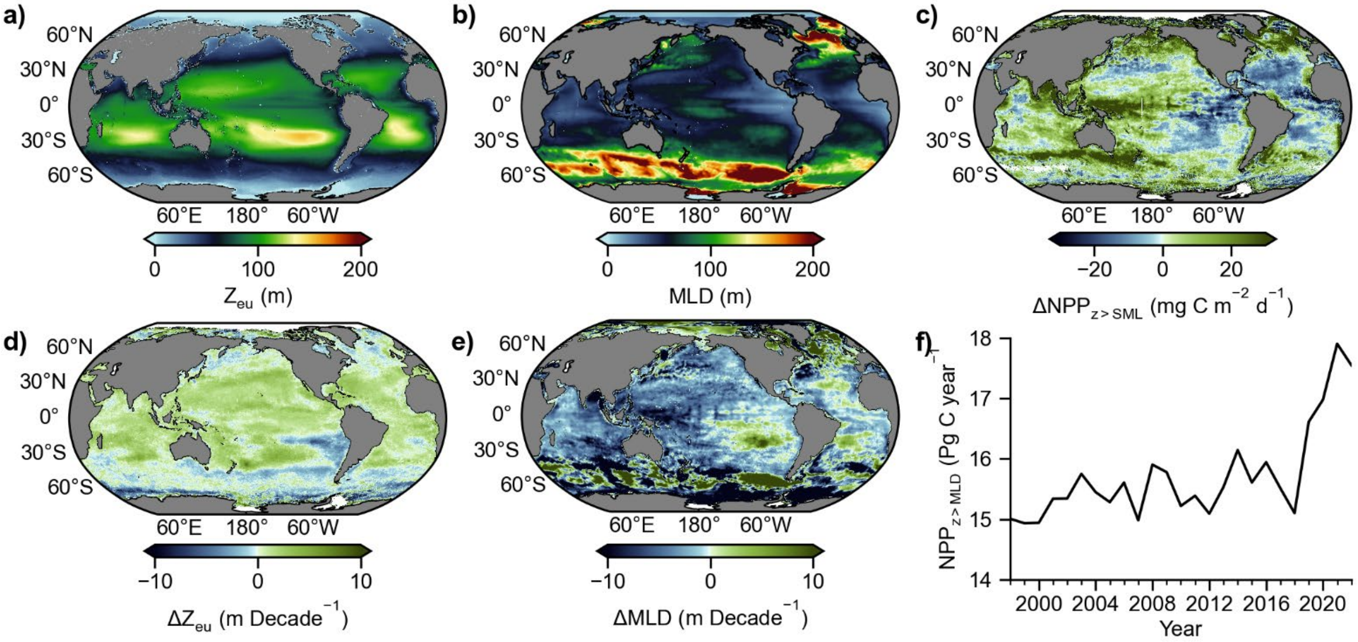

Supplementary Figure 4: **Drivers and trends of factors affecting sub-surface net primary production (NPP)**. Mean global patterns **a.** euphotic depth (Z_{eu}) and **b.** surface mixed layer depth (MLD). **c.** Decadal trends of NPP beneath the surface mixed layer ($\Delta NPP_{z>SML}$). **d.** Decadal trends in euphotic depths (ΔZ_{eu}). **e.** Decadal trends in surface mixed layer depth (ΔMLD). **f.** Yearly magnitude of NPP beneath the surface mixed layer.

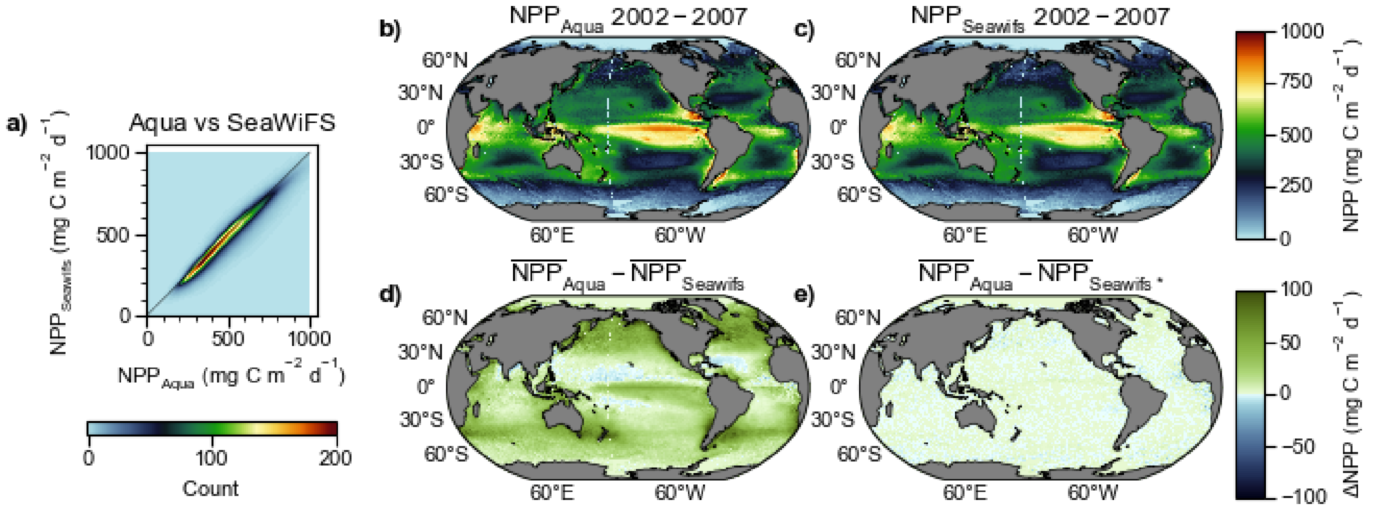

Supplementary Figure 5: Net primary production (NPP) differences between MODIS Aqua and SeaWiFS. a. Heatmap showing pixel by pixel comparison (50 km^2 resolution) of overlapping NPP measurements derived from MODIS-Aqua and SeaWiFS sensors for the period 07/2002-12/2007. Average NPP for **b.** MODIS-Aqua and **c.** SeaWiFS for the period 07/2002-12/2007. **d.** The difference between MODIS-Aqua and SeaWiFS (07/2002-12/2007). **e.** The difference between MODIS-Aqua and SeaWiFS after adjusting for monthly climatological differences between the two missions.

Supplementary Table 1: List of climate indices employed in statistical comparisons. Data is from NOAA physical sciences laboratory (https://psl.noaa.gov/data/climateindices/list/).

Name	Description
PNA	Pacific North American Index
WP	Western Pacific Index
NAO	North Atlantic Oscillation
SOI	Southern Oscillation Index
Niño 3	Eastern Tropical Pacific SST
TNA	Tropical Northern Atlantic Index
TSA	Tropical Southern Atlantic Index
WHWP	Western Hemisphere Warm Pool
ONI	Oceanic Niño Index
MEI V2	Multivariate ENSO Index Version 2
Niño 1+2	Extreme Eastern Tropical Pacific SST (0-10S, 90W-80W)
Niño 4	Central Tropical Pacific SST (5N-5S, 160E-150W)
Niño 3.4	East Central Tropical Pacific SST (5N-5S, 170-120W)
PDO	Pacific Decadal Oscillation.
TPI(IPO)	Tripole Index for the Interdecadal Pacific Oscillation.
NOI	Northern Oscillation Index
QBO	Quasi-Biennial Oscillation
Sahel Rainfall	Sahel Standardized Rainfall (20-8N, 20W-10E)
Solar Flux	Solar Flux (10.7cm)
